# Case of Forcible Tearing Away of the Uterus of a Woman Just Delivered

**Published:** 1866-08

**Authors:** 


*Case of Forcible Tearing Away of the Uterus of a woman just Delivered.*—The following case, related by Dr. Hoffmann, is a melancholy example of the evil resulting from the lack of habitual practice in ordinary midwifery, and the concomitant one of medical practitioners being called in almost exclusively to difficult labors:—A woman, aged thirty-nine, was in her ninth labor, under a midwife who gave a powder, soon after which the child was expelled. She immediately removed the placenta by drawing on the cord. Strong after-pains followed, and the midwife felt a fleshy mass in the vagina. The district surgeon found the patient pale, cold, and almost pulseless, and a dark red fleshy mass projecting from the vulva. As he could not return it, he took it for a growth or fleshy mole, and passed his hand near the mass and through an opening which he took for the os uteri. After twenty minutes' manipulation, he directed the midwife to take away the mass. Immediately this was done a loop of intestines appeared. The mass torn away was the uterus. The examination of the body showed that the lower part of the vulva, together with the perinæum as far as the anus, was torn off, and no trace of uterus, ovaries, or Fallopian tubes was found.—*Vjhrschr. f. gericktlch. Med.* 1865—*Southern Journal Medical Sciences.*

